# Toward EEG-Based BCI Applications for Industry 4.0: Challenges and Possible Applications

**DOI:** 10.3389/fnhum.2021.705064

**Published:** 2021-08-13

**Authors:** Khalida Douibi, Solène Le Bars, Alice Lemontey, Lipsa Nag, Rodrigo Balp, Gabrièle Breda

**Affiliations:** ^1^Capgemini Engineering, Paris, France; ^2^Ecole Strate Design, Sèvres, France

**Keywords:** EEG, BCI applications, BCI challenges, technology transfer, Industry 4.0

## Abstract

In the last few decades, Brain-Computer Interface (BCI) research has focused predominantly on clinical applications, notably to enable severely disabled people to interact with the environment. However, recent studies rely mostly on the use of non-invasive electroencephalographic (EEG) devices, suggesting that BCI might be ready to be used outside laboratories. In particular, Industry 4.0 is a rapidly evolving sector that aims to restructure traditional methods by deploying digital tools and cyber-physical systems. BCI-based solutions are attracting increasing attention in this field to support industrial performance by optimizing the cognitive load of industrial operators, facilitating human-robot interactions, and make operations in critical conditions more secure. Although these advancements seem promising, numerous aspects must be considered before developing any operational solutions. Indeed, the development of novel applications outside optimal laboratory conditions raises many challenges. In the current study, we carried out a detailed literature review to investigate the main challenges and present criteria relevant to the future deployment of BCI applications for Industry 4.0.

## 1. Introduction

**Figure 1:**
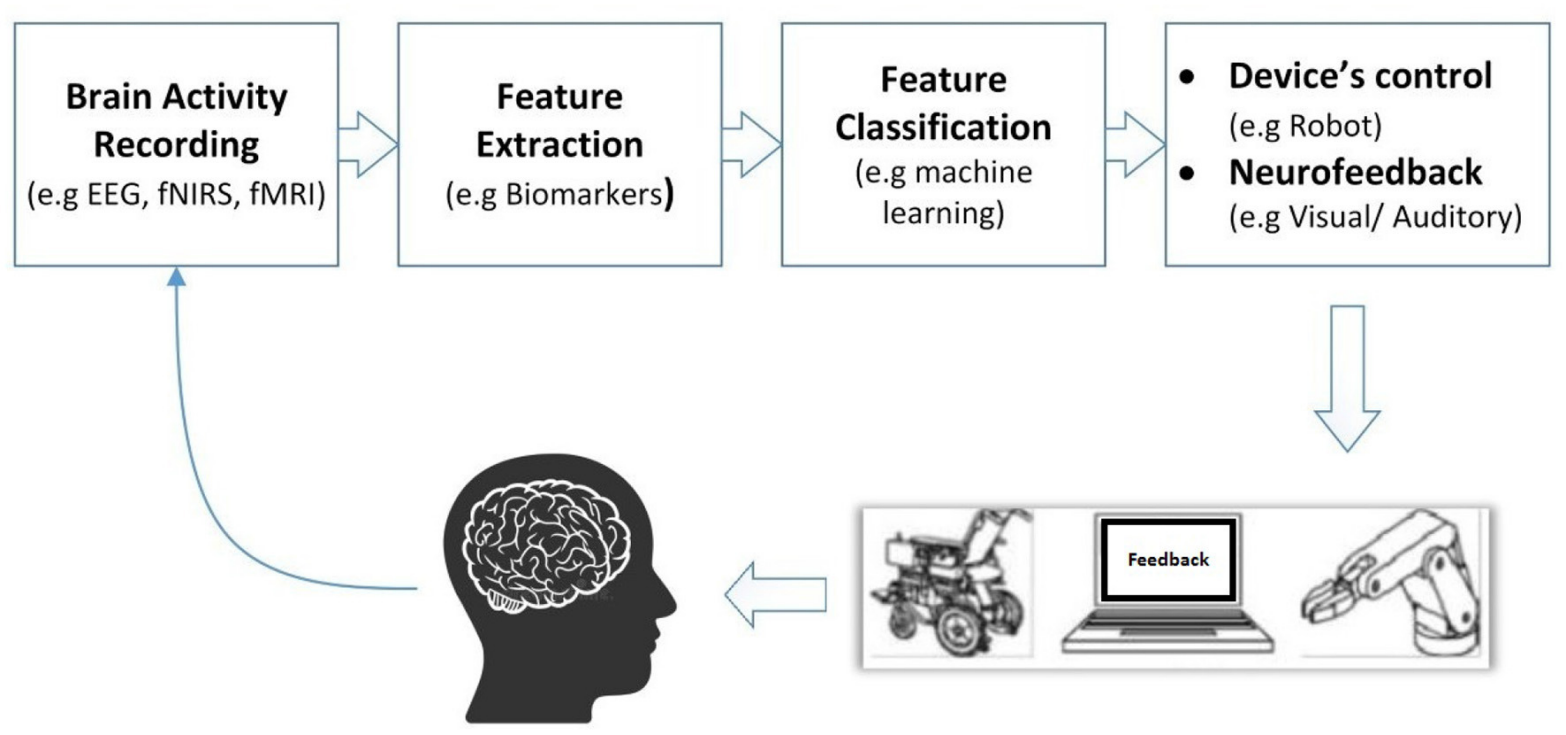
Brain-computer interface scheme.

Recent advances in neuroscience and engineering led to the development of new applications interfacing minds with machines, known as Brain-Computer Interface (BCI) technology. The origins of BCI date back to the 1960s, with Delgado (1969) who notably developed an implantable chip used to both stimulate the brain by radio and send electrical signals of the brain by telemetry, allowing the subject to move about freely. A few years later, Vidal (1973) explored the use of scalp-recorded brain signals in humans to implement a simple non-invasive BCI based on “visually evoked potentials” (see Vidal, 1973). Those experiments paved the way for the development of non-invasive BCI paradigms that made use of neuroimaging techniques as electroencephalography (EEG), magnetoencephalography (MEG), functional magnetic resonance imaging (fMRI) and functional near-infrared spectroscopy (fNIRS) (see Rao, 2013 for a comprehensive review). Indeed, by translating the recorded neural activity into digital commands via mathematical and AI methods (see Wolpaw et al., 2002) (Figure 1), BCI enables controlling external devices with the brain (e.g., Padfield et al., 2019; Khan et al., 2020), such as a computer, a robot, or an exoskeleton (e.g., Nuyujukian et al., 2018; Benabid et al., 2019; Moses et al., 2019). This ability is particularly interesting in specific contexts where voice or motor commands cannot be used (e.g., Lin et al., 2014).

Regarding the user's task and the neural patterns of interest, we can distinguish three main categories of BCI, namely, active, reactive and passive BCI (see Kögel et al., 2019). Firstly, while using an active BCI, the agent must intentionally modulate their brain activity to bring out neural characteristics that will become identifiable after mathematical processing and classification, as Motor Imagery (MI) paradigm (e.g., Salvaris, 2014). Secondly, reactive BCI relies on neural activity that is typically triggered by an external stimulus—mostly visual or auditory—and that evokes brain responses, such as P300 event-related potential (ERP) (e.g., Jin et al., 2012) or Steady State Evoked Potentials (SSEP) (e.g., Chen et al., 2017). Thirdly, passive BCI relies on brain activity which is not voluntarily modulated by the user, in order to evaluate psychological states such as drowsiness (e.g., Hongfei et al., 2011; Dehais et al., 2018), frustration, or even cognitive load (e.g., Roy et al., 2013; Myrden and Chau, 2017). More details concerning the neurophysiological underpinnings, as well as the advantages and limitations of these three BCI methods are provided in section 3.1.

Thus, in the last two decades, many types of BCI techniques and applications have emerged, especially in the clinical field where it represents a promising technology for assisting or rehabilitating neurological patients and contribute to the faster reintegration of brain-injured patients (e.g., Chaudhary et al., 2016; Verplaetse et al., 2016). However, recent advances in neuroscience and technology, especially non-invasive and portable brain imaging techniques related to EEG, have encouraged the development of novel applications outside the medical and scientific areas (e.g., Abdulkader et al., 2015; Rashid et al., 2020). Notably, one might list the following fields of education (e.g., Wegemer, 2019), entertainment (e.g., Bonnet et al., 2013; Kerous et al., 2018; Ramchurn et al., 2018; Vasiljevic and Miranda, 2020), biometrics authentification (e.g., Alariki et al., 2018; Chan et al., 2018), or even civil and military aviation (e.g., Dehais et al., 2018).

Recently, the industrial sector has also shown a growing interest in BCI (e.g., Angrisani et al., 2018), where the societal, economic and commercial impacts of this technology could be important (e.g., Van Erp et al., 2012). Indeed, since modern times, Industry has continuously seized on emerging technologies to improve its efficiency and performance and each industrial revolution has entailed deep socioeconomic changes and challenges (see Morrar et al., 2017). The last industrial revolution, also known as Industry 4.0, specifically harnessed digital technologies such as AI, big data and analytics, or the Internet of things (see Chunguang et al., 2020), which led to a constantly evolving and intelligent automation of industrial processes. However, it also raises important questions regarding environmental, ethical and human factors (see Melnyk et al., 2019). Interestingly, similar technologies are presently required to develop sophisticated BCI, notably, to efficiently process brain signals and emit commands to the connected device. Then, regarding this technological compatibility, BCI applications could theoretically constitute a potential extension of the 4th industrial revolution. Moreover, “Human enhancement” provided by BCI techniques could represent a viable way to conciliate industrial and societal concerns in a near future.

## 2. Research Goals

To this day, the use of BCI techniques in Industry 4.0 remains theoretical, being mainly explored in academic articles or exhibition demonstrators. In the following, we investigate the potential benefits of implementing BCI and how it could help to re-introduce humans within the industrial processes, by facilitating the operator's work and limiting potential risks and human errors (e.g., Jinjing et al., 2021). Indeed, technical and ethical limitations inherent in non-invasive BCI necessarily hamper the expansion of this technology within operational contexts (e.g., Rashid et al., 2020). Thus, the question of selecting the most suitable and relevant BCI technique for the industrial sector arises, especially regarding its reliability, its generalizability or its ease of use. In this context, we sought to explore the criteria that will be decisive for the potential integration of BCI in industrial settings regarding the current maturity of BCI techniques. In the current review, we attempt to answer the following question:

- Which BCI techniques are most likely to be deployed in future industrial applications?

To this end, we carried out a detailed literature review of the databases (Science direct, PubMed, IEEE, Springer, ArXiv, ResearchGate, Google Scholar, MDPI, HAL) queried with the following inclusive keywords: *BCI, Brain-computer interface, BCI and Industry 4.0, EEG-based BCI, BCI applications, BCI challenges, Assistive technology* associated to scientific studies published between 2010 and 2021. Then, we extracted recent and relevant empirical and review articles, conference proceedings, research reports related to the usage of BCI in industrial environments. Note that the exclusion criteria include “Invasive BCI techniques, non-invasive BCI for both clinical applications and non-industrial fields.” In addition, all the references that are provided in the following sections serve as recent examples for the identified BCI applications for Industry 4.0.

## 3. Results and Discussion

### 3.1. BCI Applications for Industry 4.0

Theoretically, the deployment of BCI applications in Industry 4.0 could contribute to put the operator back at the center of industrial processes. The possible industrial applications could be categorized as follows: (1) safety at work, (2) adaptive training and (3) device's control (e.g., Tamburrini, 2014; Balderas et al., 2015; Oztemel and Gursev, 2018).

#### 3.1.1. Safety at Work: *Passive BCI*

Recently, there has been a growing interest in using EEG-based BCI as a potential solution allowing to reduce safety risks, while enhancing productivity and improving decisions in operators and managers (e.g., Villalba-Diez et al., 2019). Regarding technical aspects, this application would rely on the use of passive BCI. Notably, the decomposition of EEG signal into frequency bands represents a convenient way to identify the user's neurocognitive condition. For instance, many studies have shown that the spectral power in alpha (8–13 Hz) and theta (4–7 Hz) bands increase when a person feels fatigued (e.g., Craig et al., 2012; Dehais et al., 2018). Such modulation can therefore constitute a first-rate indicator of the user's arousal state (e.g., Zhang et al., 2017). Changes in theta rhythm in frontal sites and alpha rhythm in parietal sites (e.g., Borghini et al., 2013), could also indicate a state of cognitive overload, which has been linked to reduced performances in complex tasks (e.g., Aricò et al., 2018). Thus, by allowing user-state monitoring, passive BCI could notably limit or prevent safety risks and human errors without requiring any particular effort from the user. Theoretically, this passive aspect makes it potentially usable in multitasking contexts and does not induce additional fatigue. However, passive BCI is subject to an important inter-individual variability. Moreover, EEG band frequencies must be carefully analyzed because similar spectral power patterns could be associated to several mental states (e.g., Aricò et al., 2018).

Then, the neurofeedback provided by passive BCIs could reinforce safety at work by preventing agents from committing dangerous errors due to drowsiness or cognitive overload for instance (e.g., Villalba-Diez et al., 2019). In fact, some industrial sectors—including among others, manufacturing, quality control or pharmaceutical industry—require operators to carry out a large number of repetitive and sensible actions that directly depend on the operator's neurocognitive states (e.g., Villalba-Diez et al., 2019). In this context, EEG-based BCI could allow to monitor operators' mental states like fatigue, stress, or loss of vigilance which can be critical during dangerous activities. In particular, fatigue monitoring is more considered as a valuable tool in repetitive and automatic tasks such as driving, piloting or quality control (e.g., Zhang et al., 2017; Huang et al., 2019). For this reason, some solutions that integrate EEG captors within worksite helmets (e.g., Li et al., 2014; Barkallah et al., 2015) or under headwears (e.g., Zhang et al., 2017) have already been proposed to warn users whenever a critical drowsiness threshold is reached.

#### 3.1.2. Adaptive Training: *Passive BCI*

Another emerging BCI application—also relying on passive BCI technique—concerns adaptive training, which might reinforce the learning process of complex industrial procedures. This neurofeedback approach is already used in clinical settings, to support learning or rehabilitate attention in children with neurodevelopmental disorders (e.g., Papanastasiou et al., 2020). Indeed, such monitored training would allow boosting attentional processes while adapting task difficulty according to cognitive load or vigilance, to optimize learning and prevent frustration. In this context, Huang et al. (2019) proposed to combine BCI with other technologies such as Virtual Reality (VR) and/or Augmented Reality (AR) to make the learning task even more immersive and efficient. Similarly, Nisiforou (2013) proposed the use of eye tracking coupled with EEG to assess and evaluate students' cognitive dimensions.

#### 3.1.3. Device's Control: *Reactive* and *Active BCI*

Besides monitoring applications, another potential industrial use case concerns cobots or machine' s control. Both active and reactive BCI paradigms could be relevant for this kind of application. Regarding active BCI, motor imagery (MI-BCI) is the most commonly used paradigm. During this task, the user is typically required to imagine specific movements (e.g., for limb), that allows controlling an external device in the same way (e.g., a robot, an exoskeleton or an avatar etc.). Indeed, imagining a movement typically produces neural activity that is spatiotemporally similar to the activity generated during actual movement, but smaller in magnitude (see Wolpaw et al., 1991; Pfurtscheller et al., 1997; McFarland et al., 2000). Although this method is particularly promising in the context of motor disability, its main drawbacks stem from some limitations of the EEG method itself. Notably, due to a low spatial resolution, it is not possible to localize accurately activation sources within the same hemispheric sensorimotor cortex, which prevents the reliable identification of fine motor movements (e.g. distinguishing a movement of the whole arm from a movement of the sole hand). This generally limits the number of potential and reliable orders to 4 (e.g., Schlögl et al., 2005). Another disadvantage of the MI paradigm relies on the EEG low signal/noise ratio. Indeed, due to its non-invasive nature, EEG recordings also contain irrelevant, non-brain signals like environmental electromagnetic artifacts or peripheral nervous transmissions. In addition, this technique requires a long training phase to be properly mastered (e.g., Vidaurre et al., 2011) that can last several days or weeks and which is incompatible with many industrial contexts. Then, even after a substantial training phase, more than 30% of individuals would remain BCI illiterate (e.g., Ahn et al., 2014; Lee et al., 2019), by staying unable to control the device. Finally, motor imagery requires an intense concentration from the user and is incompatible with other “real” movements that would interfere with the thought command and ultimately, with the BCI accuracy. Thus, MI paradigms prevents the user from performing other tasks at the same time and necessarily generates fatigue (e.g., Talukdar et al., 2019) which makes its use rather inflexible. Despite all these limitations, MI training session design is of vital importance for clinicians planning to implement interventions adapted to participant health status, age and gender. For that, numerous studies are attempting to overpass these limitations—notably regarding the problematic user-training phase—by proposing some guidelines that could be useful to improve this critical dimension (e.g., Schuster et al., 2011; Jeunet et al., 2016)

Regarding reactive BCI, the two most widely used and reliable EEG markers are the P300 and SSVEP (steady-state evoked potentials). P300 is a positive event-related potential that is apparent whenever the user has noticed an unexpected or a rare visual or auditory event (e.g., Walter et al., 1964; Donchin and Smith, 1970). Although associated to a fast training, this technique remains very sensitive to surrounding noise and motor artifacts (e.g., Chamola et al., 2020), preventing its use in a noisy or multitasking context. In addition, a single command requires the user to focus their attention on several consecutive events, including non-relevant (non-rare) and relevant (rare and unexpected) ones, which necessarily decreases the system's speed (e.g., Lotte et al., 2015) while being costly in terms of attentional processes (fatigability).

As regards SSVEPs, the distinct potential commands are displayed via a visual interface (e.g., screen or AR glasses), icons that flicker at distinct frequencies (e.g., 10 Hz) represent different options. Then, while the user is focusing on one flickering option, visual neurons (i.e., from the primary visual cortex) are synchronously discharging at the same rate, which will ultimately allow the user's choice to be identified with classification algorithms (e.g., Middendorf et al., 2000; Faller et al., 2010). Interestingly, SSVEP-BCI is less prone to inter-individual differences, which enhances its accuracy (e.g., Lotte et al., 2015) and reduces its illiteracy rate (e.g., Lee et al., 2019). Moreover, it does not require a long training phase (e.g., Guger et al., 2012) while the latency between the neural command and the command execution can potentially be lower than in other BCI paradigms. However, similarly to the P300 paradigm, extended use can induce significant fatigue, due to the required active concentration on stimuli. Another disadvantage of reactive BCI is the need to use external stimuli to allow the agent to make a choice. The exerted control is therefore limited to the presented options and is not strictly endogenous. Moreover, it requires an additional interface, such as a screen, which decreases its portability (e.g., Cecotti et al., 2010). Regarding this last limitation, recent works have attempted to create new interface designs and stimuli that reduce fatigue and discomfort, to promote a daily and long-term use of this BCI paradigm (see Baek et al., 2019).

In industrial settings, an SSVEP-BCI combined with AR glasses could facilitate making certain tasks hands-free (and therefore, replace buttons/joysticks) for operators who control machines (e.g., Angrisani et al., 2018, 2020). Looking further ahead, one might also imagine that a MI-BCI could eventually allow to quickly take over control of transport vehicles in case of emergency braking. In this scenario, BCI must be sufficiently advanced to allow a reliable transmission of the “thought” braking command to the mobile device. It should also be faster than our peripheral nervous transmission to become valuable regarding accidents prevention, which is far from being the case at present (e.g., Royer et al., 2010; Kim and Lee, 2017; Georgescu et al., 2020).

### 3.2. Specifications and Limitations

Regardless the potential benefits that BCI could bring to Industry and besides the actual weaknesses of EEG-based BCI, it is also necessary to consider its current ethical, ergonomic and technical limitations, before any operational development or usage.

The first limitation relates to **ethics** and acceptability that must be further questioned and regulated regarding the individual and societal impacts that industrial BCI applications might have. Among other things, industrial BCI must ensure data confidentiality and security given the sensible and personal nature of recorded physiological signals (e.g., Burwell et al., 2017). In addition to the operator consent, individual data must be locally stored and processed. Then, the relevant extracted information must be accessible to the sole concerned operators. To be acceptable to the end users, the BCI system must provide a real improvement of work conditions and/or safety by limiting the risks in dangerous conditions, such as passive BCI. Presently, non-invasive BCI for device control (active and reactive BCI) remains too immature to get easily used and adopted by the agents. According to Burwell et al., 2017, the need for regular and challenging training sessions (e.g, Motor Imagery) may impose physical, emotional, and financial burdens on the user (e.g., Fenton and Alpert, 2008) and it may require more cognitive planning and attention than a user can achieve on a regular basis, leading to frustration (e.g., Glannon, 2014).

Another crucial requirement for an effective adoption by end-users concerns **ergonomics** (e.g., Li et al., 2014). More precisely, BCI solutions must be non-invasive; comfortable to wear; portable and not bulky to allow mobility in different areas; non-tiring for the user; multitasking-compatible to allow usual tasks without limitations; inexpensive in terms of training time and dedicated resources. On the one hand, passive BCIs are currently more suited to the criteria of portability, non-fatigability and multitasking since they do not require external stimuli and devices (e.g., AR glasses or monitor) and they do not require to perform a particular cognitive task, in comparison with active and reactive paradigms (see Wolpaw et al., 2002; Rao, 2013). On the other hand, the training cost is particularly important in active BCI, while it is less important in reactive and passive BCI (e.g., Cecotti, 2010; Jeunet et al., 2017; Myrden and Chau, 2017).

Moreover, BCI **technical** specificities must be considered to ensure: (1) *reliability*—which depends on classifications' *accuracy*—(2) *reactivity* in terms of response time and (3) *flexibility* to adapt to context and individual differences (e.g., Rashid et al., 2020). In other words, an ideal BCI solution must be able to interpret an operator's neural signal, by minimizing classification errors and training time, while increasing the information transfer rate (ITR) and its generalizability of use (e.g., Rashid et al., 2020). According to literature, reliability and flexibility appear to be higher in reactive BCI and particularly in SSVEP-based BCI (e.g., Chen et al., 2014), relative to active and passive paradigms. Flexibility appears lower in active paradigms, with a high illiteracy rate, in comparison with reactive and passive paradigms (e.g., Lee et al., 2019). Reliability depends on the quality of the collected signal and the relevance of AI algorithms applied. Besides, some neurophysiological markers used are more or less resistant to surrounding noise and should be carefully selected with a reduced latency. In addition, a large-scale deployment BCI requires adaptability and flexibility to make it usable and equally reliable for a large number of users.

Based on the advantages and disadvantages of each BCI application described previously (section 3.1), Table 1 summarizes the estimated levels of adequacy between the main industrial criteria (section 3.2) and the actual state-of-the-art EEG-based BCI, in the light of potential applications. More specifically, we have compared the three main non-invasive BCI paradigms and their potential industrial applications considering the most important criteria related to ethics, ergonomics & UX, and technical.

**Table 1:** Levels of adequacy between industrial and technical BCI requirements based on the EEG, regarding potential industrial applications.

		**Active BCI**	**Reactive BCI**		**Passive BCI**	

		**Motor imagery**	**P300**	**SSVEP**	**Fatigue monitoring**	**Cognitive load monitoring**
	Industrial applications	Device control	Device control	Device control	Safety, training	Safety, training
Ethics	Acceptability	++	++	++	++	+
Ergonomics and user experience	Portability	+++	++	++	+++	+++
	Fatigability	+	++	++	+++	+++
	Multitasking	+	+	++	+++	+++
	Training/calibration	+	++	+++	++	+
Technical	Reliability	+	++	+++	++	++
	Rapidity	+	+	++	++	++
	Flexibility	+	+	++	+++	+++

The level of adequacy is ranked as follow: “+” rating represents a low level of match between the industrial requirement and the BCI technique, while “++” and “+++” means an intermediate and a high level of suitability, respectively.

## 4. Conclusions and Perspectives

BCI is an emerging technology that enables to decode brain activity and translate it into a set of actions reflecting the user's intention, mental state, and even emotions. Numerous public and private actors are envisioning the deployment of BCI in industrial settings in a near future (e.g., Sujatha Ravindran et al., 2020). In the present paper, we summarized the potential applications, key success factors and the most advanced EEG-based BCI paradigms for Industry 4.0. Currently, none of the EEG-based BCI evaluated fits ideally to all the essential industrial criteria we have established based on ethical, ergonomic and technical factors. However, SSVEP-based BCI represents a highly promising technology for device control, while fatigue monitoring appears particularly interesting and appropriate to prevent damaging errors and safety risks in dangerous contexts, or optimize training upstream of these critical situations. With the dramatic rise of EEG-based BCI studies—regarding material, associated algorithms (machine learning, deep learning etc.) or even psychological aspects of BCI—we believe that the large-scale deployment of BCI applications in the Industry is a matter of years. Thus, the ethics and rules related to BCI applications in industrial settings need to be carefully defined to pave the way to effective use.

## 5. Study Limitations and Further Work

The authors are aware that the present results should be interpreted with caution and some important limitations deserve to be mentioned. Firstly, the envisioned industrial applications of BCI are still at the first stages of research and development. Thus, the development of reliable and ethical BCI solutions adapted to end-users in industrial contexts will necessarily take several years. Secondly, research focussing on BCI users' experience remains extremely rare and further studies must explore this important dimension. Though the users' experience is currently strongly related to technical specificities of BCIs solutions, a user-centered approach similar to the one proposed by Kubler et al. (2014) must be systematically used in future research before considering any large-scale deployment of such neurotechnology.

## Author Contributions

KD conceived the initial idea, conducted the research, and wrote the first draft with AL. GB reviewed the article's structure. SL contributed with BCI paradigms relevant to Industry 4.0, performed the synthesis, and perspectives with KD. RB and LN contributed, with other authors, to the final proofreading and commented on the draft. All authors have read and approved the final paper.

## Conflict of Interest

The authors declare that the research was conducted in the absence of any commercial or financial relationships that could be construed as a potential conflict of interest.

## Publisher's Note

All claims expressed in this article are solely those of the authors and do not necessarily represent those of their affiliated organizations, or those of the publisher, the editors and the reviewers. Any product that may be evaluated in this article, or claim that may be made by its manufacturer, is not guaranteed or endorsed by the publisher.
